# Erectile function in circumcised and uncircumcised men in Lusaka, Zambia: A cross-sectional study

**DOI:** 10.4102/phcfm.v7i1.766

**Published:** 2015-06-26

**Authors:** Evans Chinkoyo, Michael Pather

**Affiliations:** 1 Faculty of Medicine and Health Sciences, Department of Interdisciplinary Health Sciences, Division of Family Medicine and Primary Care, University of Stellenbosch, South Africa; 2 Chipata Level 1 Hospital, Lusaka, Zambia

## Abstract

**Background:**

Evidence from three randomised control trials in South Africa, Uganda and Kenya showing that male circumcision can reduce heterosexual transmission of human immunodeficiency virus (HIV) infection from infected females to their male partners by up to 60% has led to an increase in circumcisions in most African countries. This has created anxieties around possible deleterious effects of circumcision on erectile function (EF).

**Aim:**

To compare EF in circumcised and uncircumcised men aged 18 years and older.

**Setting:**

Four primary healthcare facilities in Lusaka, Zambia.

**Methods:**

Using a cross-sectional survey 478 participants (242 circumcised and 236 uncircumcised) from four primary healthcare facilities in Lusaka, Zambia were asked to complete the IIEF-5 questionnaire. EF scores were calculated for the two groups, where normal EF constituted an IIEF-5 score ≥ 22 (out of 25).

**Results:**

Circumcised men had higher average EF scores compared to their uncircumcised counterparts, (*p* < 0.001). The prevalence of erectile dysfunction was lower in circumcised men (56%) compared to uncircumcised men (68%) (*p* < 0.05). EF scores were similar in those circumcised in childhood and those who had the procedure in adulthood, (*p* = 0.59). The groups did not differ significantly in terms of age, relationship status, smoking, alcohol and medication use. A statistically significant difference was observed in education levels, with the circumcision group having higher levels of education (*p* < 0.005).

**Conclusion:**

The higher EF scores in circumcised men show that circumcision does not confer adverse EF effects in men. These results suggest that circumcision can be considered safe in terms of EF. A definitive prospective study is needed to confirm these findings.

## Abstrait

**Fonction érectile chez les hommes circoncis et non circoncis à Lusaka, Zambie: Une étude transversale.**

**Contexte:**

Les preuves des trois essais contrôlés randomisés en Afrique du Sud, en Uganda et au Kenya montrent que la circoncision masculine peut réduire de 60% la transmission hétérosexuelle de l’infection VIH des femmes infectées à leur partenaire masculin ; cela a eu pour résultat une augmentation des circoncisions dans la plupart des pays africains. Mais cela a aussi causé des craintes sur les effets nuisibles possibles de la circoncision sur la fonction érectile (FE).

**Objectif:**

Comparer la FE chez les hommes circoncis et non circoncis de 18 ans et plus.

**Lieu:**

Quatre établissements de santé primaire à Lusaka, Zambie.

**Méthodes:**

Au cours d’une étude transversale on a demandé à 478 participants (242 circoncis et 236 non circoncis) de quatre établissements de soins primaires à Lusaka, Zambie de remplir le questionnaire IIEF-5. Les résultats des deux groups ont été calculés, et on a constaté que la FE normale avait un résultat IIEF-5 ≥ 22 (sur 25).

**Résultats:**

les résultats moyens de FE des hommes circoncis étaient plus élevés que ceux des non circoncis, (*p* < 0.001). La fréquence de dysfonctionnement érectile était moindre chez les hommes circoncis (56%) que chez les non circoncis (68%) (*p* < 0.05). Les résultats de la FE étaient semblables chez ceux qui avaient été circoncis dans leur enfance et ceux qui l’avaient été à l’âge adulte, (*p* = 0.59). Les groupes ne différaient pas énormément selon leur âge, leur situation sexuelle, le fait qu’ils fument ou qu’ils boivent de l’alcool et la prise de médicaments. On a observé une différence importante statistiquement dans le niveau d’éducation, où le groupe de circoncis avait un plus haut niveau d’éducation (*p* < 0.005).

**Conclusion:**

Le résultat plus élevé de la FE chez les homes circoncis montre que la circoncision ne produit pas d’effets néfastes de la FE chez les hommes. Ces résultats suggèrent que la circoncision peut être considérée comme sans danger pour la FE. Il faudra faire une étude prospective définitive pour confirmer ces résultats.

## Introduction

Male circumcision, defined as the surgical removal of the foreskin, has been practised for various reasons since time immemorial. In some cultures it is practised as a rite of passage and is accompanied by a period of initiation where newly circumcised boys are given life skills and lessons on how to live as responsible men later on in life. In parts of the world where circumcision is practised for religious purposes, this usually signifies a covenant with God and is performed in the neonatal period, or at some other time during childhood. Circumcision also has a role in medicine as treatment for some penile conditions and as a means of reducing the chance of acquiring some sexually transmitted infections and other non-communicable diseases of the penis.^1,2^

Whilst benefits of male circumcision are well documented, questions about its effects on erectile function (EF) continue to be asked. Most studies that have been conducted to explore the relationship between male circumcision and EF have yielded conflicting results.^3,4,5,6^ This study aimed to compare EF in circumcised and uncircumcised men in Lusaka, Zambia.

### Study rationale and motivation

The fear of developing sexual problems following circumcision has resulted in a lot of myths around the procedure. Studies conducted so far have failed to provide consensus on this issue. The evidence generated from this study will help people to understand what happens to them after circumcision, and will help them make informed choices regarding this procedure. The evidence will also help to inform international efforts during implementation of country circumcision programmes for prevention of HIV infection.

### Aim and objectives

The aim of this study was to compare EF in circumcised and uncircumcised adult men aged 18 years and above in Lusaka, Zambia.

The research question in this study was ‘Is there a difference in (erectile function) EF between circumcised and uncircumcised men in Lusaka, Zambia?’

The objectives of the study were as follows:

- To determine the prevalence of erectile dysfunction (ED) amongst circumcised and uncircumcised men aged 18 years and above.
- To compare the prevalence of ED in circumcised and uncircumcised men aged 18 years and above.
- To determine whether the age at which circumcision was performed in study participants had any effect on EF in adulthood.
- To make recommendations on how to respond to concerns regarding EF following circumcision.

The aims and objectives of the study were derived from the hypothesis that there was no significant difference in EF between circumcised and uncircumcised males.

## Literature review

Male circumcision is increasingly being accepted as an additional viable strategy for the prevention of HIV transmission from infected females to their uninfected male partners. Three randomised clinical trials in South Africa, Kenya and Uganda have demonstrated that male circumcision can provide partial protection for heterosexual men against HIV infection from infected female sexual partners.^7^ This has prompted Ministries of Health in most countries hard-hit by the HIV pandemic to consider male circumcision as an additional strategy for prevention of HIV infection. In Zambia a strategy to circumcise up to 2.5 million males between the ages of 13 and 39 years by 2020 has been launched and measures put in place to ensure its success.^8^ Most countries in sub-Saharan Africa have also introduced plans to circumcise up to 80% of eligible males in their populations.^9^ The expected outcome of these interventions is a reduction in new HIV infections.

Whilst emphasis is currently on prevention of HIV infection, there are several ongoing debates around the safety, relevance and human rights aspects of male circumcision.^10,11^ Some of these discussions are centred around children,in view of their inability to consent to the procedure on their own and having to rely on adults to make decisions on their behalf.

Questions are also being asked about the effect of circumcision on sexual function and the ability of a circumcised man to initiate and maintain a satisfactory erection for normal sexual intercourse. Normal sexual function requires intact genitalia, good blood flow to pelvic organs, an intact neuro-endocrine system and a healthy psychological state.^12^ Male circumcision interferes with the integrity of the genitalia by removing the foreskin together with its nerves and blood vessels. This partial denervation of the penis and the subsequent keratinisation of the exposed glans can potentially cause sensory changes resulting in altered ability to experience tactile stimulation, which is necessary for initiation and maintenance of a penile erection.

There have been several attempts to explore the relationship between male circumcision and sexual function, but they have yielded disparate results. In a study of the effect of circumcision on EF, penile sensitivity, sexual activity and satisfaction, Fink et al.^4^ observed, amongst other findings, that adult circumcision appeared to result in worsened erectile function and decreased penile sensitivity. Several studies of this nature have been published and yielded similar results.^13,14^ Other studies also reported reduced glans sensitivity following circumcision, but without any difference in EF.^15,16^ A review of international evidence for benefits and risks of infant circumcision^17^ concluded that male circumcision had no adverse effect on sexual function, penile sensation or satisfaction. In a randomised controlled study conducted in Uganda^6^ circumcision did not appear to have any adverse effects on sexual function and satisfaction in men. However, this study had limitations in that blinding was not possible and therefore there was a possibility of both interviewer and reporting bias by participants.

Another study looking at the effect of circumcision on male sexual function in Kenya^7^ also observed that circumcision did not have clinically important adverse effects on male sexual function in sexually active adults who underwent the procedure. This same result was echoed by systematic reviews and a meta-analysis of scientific literature on this subject which concluded that male circumcision has no adverse effect on sexual function, sensitivity, sexual sensation, or satisfaction.^18,19^

This lack of consensus at international level called for local exploration of the subject in order to establish whether similar results could be reproduced in Zambia, a country with a different cultural context. Since there had not been any formal studies to establish the prevalence of ED amongst Zambian men, the survey aimed to simultaneously measure the prevalence of ED amongst circumcised and uncircumcised men in order to compare the results. The study also sought to determine whether there was any difference in EF in those circumcised in childhood compared to those circumcised in adulthood.

## Research methods and design

### Study design

This was a descriptive cross-sectional survey. This study design was adequate for the main aim and objectives of the survey.

### Setting

The study was conducted in outpatient departments of four primary healthcare facilities in Lusaka, Zambia between 01 June 2013 and 30 September 2013. The four healthcare facilities were the Matero, George, Kanyama and Chilenje Health Centres.

### Study population

The population of interest for this study comprised circumcised and uncircumcised sexually active males older than 18 years living in Lusaka, Zambia.

The survey included all sexually active men older than 18 years who were visiting the study sites for various reasons, and those who had responded to requests to participate in the study (e.g. patients with minor ailments, men accompanying patients, employees and their partners, men previously circumcised at the centres).

Exclusion criteria were males younger than 18 years, men with mental and physical conditions that would have made it difficult for them to participate in the study (e.g. clinical depression, psychosis, serious physical illness, alcohol or other drug intoxication), lack of sexual experience, and refusal to participate in the study.

### Sample size and sampling method

A convenience sample of an equal number of circumcised and uncircumcised men totalling a minimum of 460 individuals was chosen for the study. The sample size was calculated based on the assumption of 25% disease in the uncircumcised group and 37.5% in the circumcised one; two-sided confidence level 95%, power 80%. A total of 242 circumcised and 236 uncircumcised men took part in the study. The population that was accessible to the study consisted of all eligible adult males visiting Chilenje, Matero, Kanyama and George Health Centres during the study period. Since all the study sites also serve as circumcision centres, circumcision records with contact details dating back the last few years were also used as sampling frames to recruit willing participants into the study. Such candidates were non-randomly contacted by telephone with requests to participate. Participants were also requested to encourage their peers and family members to participate.

The four participating sites are scattered across Lusaka and generally receive people from different sections of society, and can therefore be reasonably considered representative of the population of interest. Chilenje Health Centre is located in a peri-urban township that has relatively higher education levels and income per household than the Kanyama and George compounds. Matero community falls somewhere in between Chilenje and the Kanyama and George compounds in terms of socio-economic development. The sampling frame was also representative of males who had undergone circumcision under the programme that stimulated interest for this study.

### Data collection and measurement methods

Adult men visiting Matero, Kanyama, George and Chilenje Health Centres during the study period were approached with the request to participate in the survey. These included circumcised and uncircumcised male patients, employees, partners of female employees, men previously circumcised at the centres and others referred by participants themselves. Eligibility for the survey was ascertained first and the purpose of the study explained before requesting them to participate in the study. Those who agreed to participate were given participant information sheets containing details of the study. They were assured of confidentiality and each one of them gave written informed consent before enrolling into the study. All participants were given the freedom to decline to participate and to withdraw from the study at any point without fear of any reprisals. They were then handed the IIEF-5 questionnaire with seven demographic questions to complete.

The measure used in this study (IIEF-5 questionnaire)^20^ is a well-known, abridged version of the International Index of EF questionnaire (IIEF).^21^ The IIEF-5 questionnaire was administered to study participants as part of a structured interview during which other demographic data were also captured, for example age, level of education, relationship status, smoking habits, alcohol use, and use of medications, including sexual enhancers. This questionnaire comprises four questions from the EF domain and one question from the intercourse satisfaction domain of the IIEF. Each of the five items of the questionnaire can be scored from a minimum of 1 to a maximum of 5. The IIEF-5 score20 is the sum of the ordinal responses to the five items in the questionnaire. The following are the possible scores with their interpretations: 22–25 – no ED, 17–21 – mild ED, 12–16 – mild-to-moderate ED, 8–11 – moderate ED, and 5–7 – severe ED.

The IIEF-5 has been validated in several cultures and languages, and has been shown to have good reliability and discriminant validity.^22,23,24^ Participants were divided into two groups, that is circumcised and uncircumcised. All participants received the same IIEF-5 questionnaire, and those who could not read and/or write were assisted to answer it in private. They were assured of confidentiality, and each one of them was only surveyed once.

### Data and statistical analysis

Data were captured on paper-based questionnaires which were kept in locked cabinets. These data were subsequently entered into an Excel data set on a password-protected computer.

IIEF-5 scores were analysed to assess EF, whilst demographic data were evaluated to screen for confounding factors. Chi-square tests were used to examine differences in some categorical variables (alcohol use, cigarette smoking, relationship status and education level), whilst Mann-Whitney *U*-tests were used for comparison of the two groups by age, medication use and EF scores. Calculated probabilities of < 0.05 were considered to be significant and are quoted to three decimal places. All other statistical results are quoted to two decimal places.

### Ethical considerations

This study was conducted with strict adherence to the ethics standards of the University of Stellenbosch Human Research Ethics Committee and the University of Zambia Biomedical Research Ethics Committee, and in accordance with the Helsinki Declaration of 1975, as revised in 2008.

## Results

There were 478 participants in this study, 242 in the circumcised group and 236 in the uncircumcised one. There were very few differences between the two groups of participants in terms of age, relationship status, alcohol use, smoking and medication use. However, significant differe nces were observed in participants’ levels of education and EF scores.

### Erectile function evaluation

**FIGURE 1:**
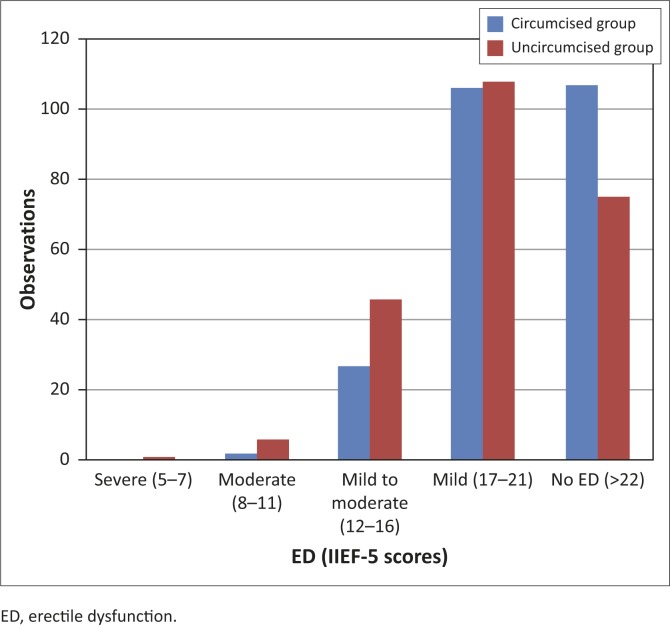
ED scores in the circumcised and the non-circumcised groups, variable IIEF-5 scores.

Figure 1 depicts IIEF-5 scores by group. Most of the scores for both groups (92%) were between 16 and 25, that is in the mild to no ED range.

### Comparison of erectile function scores

**FIGURE 2:**
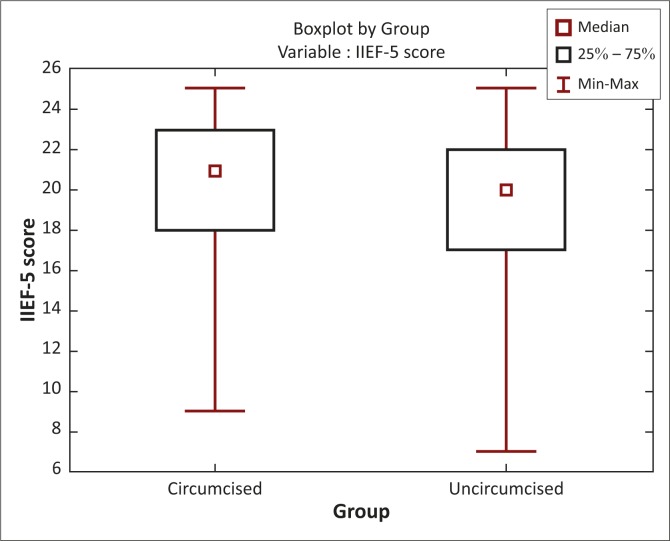
Boxplot by circumcised and uncircumcised group plotted against IIEF-5 scores.

Figure 2 shows IIEF-5 scores plotted against groups. The median in circumcised men was higher than in uncircumcised men. The two groups showed statistically significant differences to each other, with higher average scores observed in the circumcised group (*U* = 23062.50, *Z* = 3.64, *p* < 0.001).

### Prevalence of erectile dysfunction

The prevalence of ED in all participants surveyed was around 62%. Amongst circumcised participants 44% registered normal EF compared to 32% in the uncircumcised group. These results imply that 56% of circumcised and 68% of uncircumcised participants had varying degrees of ED (Table 1).

**TABLE 1:** Two-way summary table of observed frequencies of IIEF-5 scores ≥ or < 22.

IIEF-5 score	Circumcision group *n* (%)	Uncircumcised group *n* (%)
≥ 22	107 (44.2%)	75 (31.8%)
< 22	135 (55.8%)	161 (68.2%)
**Total**	**242 (100%)**	**236 (100%)**

#### Comparison of erectile dysfunction in the two groups

The observed difference in the prevalence of ED in the two groups was statistically significant (χ^2^ [*N*182] = 7.83, df = 1, *p* < 0.05). More participants in the circumcised group had normal EF than participants in the uncircumcised group.

### Education level

The circumcised group had significantly more participants with higher education levels than the uncircumcised group (χ^2^ [*N*478] = 19.05, df = 6, *p* < 0.005) (Table 2).

**TABEL 2:** Two-way summary table of observed frequencies of level of education.

Education level	Group-circumcised	Group uncircumcised	Row-totals
None	3	3	6
%	50	50	-
Primary (Grades 1–7)	20	29	49
%	40.82	59.18	-
Junior Secondary (Grades 8–9)	26	52	78
%	33.33	66.67	-
Senior Secondary (Grades 10–12)	76	70	146
%	52.05	47.95	-
College certificate or diploma	96	70	166
%	57.83	42.17	-
Undergraduate degree	20	9	29
%	68.97	31.03	-
Postgraduate degree	1	2	3
%	33.33	66.67	-
Missing data	0	1	1
%	0	100	-
**Totals**	**242**	**236**	**478**

### Relationship between age at circumcision and erectile function

Table 3 shows the distribution of participants who were circumcised in childhood and those circumcised in adulthood. The prevalence of ED was around 58% and 56% in those circumcised in childhood and adulthood respectively. These results did not show any statistically significant difference between the two subgroups (χ^2^ [*N*242] = 0.29, df = 1, *p* = 0.59).

**TABLE 3:** Age at circumcision.

Category	Number	%
Circumcised in childhood (< 18 years old)	107	44.2
Circumcised in adulthood (> 18 years old)	135	55.8
**Total**	**242**	**100**

## Demographic characteristics

### Age of participants

**FIGURE 3:**
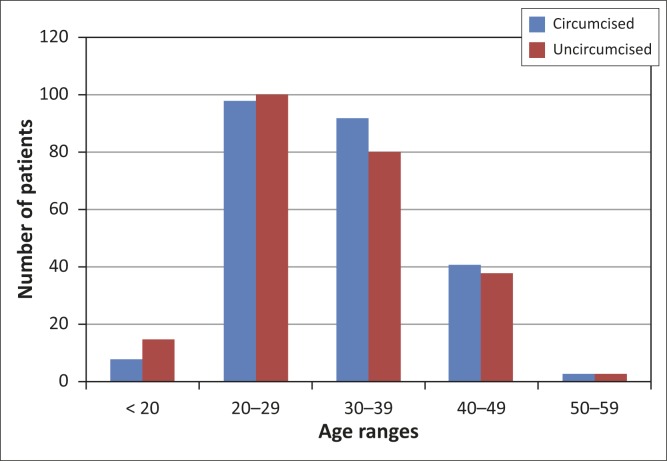
Age ranges in the circumcised and uncircumcised group.

A review of participants’ age ranges was conducted for all 478 patients in the study (Figure 3). The mean ages of the two groups did not differ significantly (*U* = 26944.50, *Z* = 1-066976, *p* = 0.286).

### Relationship status

The majority of participants were either married (58%) or single (37%). About 3% were divorced, whilst those who were separated from their partners and widowers accounted for 1% each. No statistically significant difference was observed between the two groups in this respect (χ^2^ [*N*478] = 6.69, df = 4, *p* = 0.153).

### Alcohol use

About 53% of participants in the circumcision group admitted to using alcohol, whilst in the uncircumcised group 51% reported alcohol use. No statistically significant difference was found between the two groups in the use of alcohol (χ^2^ [*N*247] = 0.10, df = 1, *p* = 0.758).

### Smoking

Smokers represented 18% and 23% in the circumcised and uncircumcised groups respectively. There was no statistically significant difference in this category between the two groups (χ^2^ [*N*97] = 1.41, df = 1, *p* = 0.235).

### Medication use

In the circumcised group 7% reported use of antihypertensive drugs, whilst less than 1% indicated use of anti-diabetic medications. Almost 2% and less than 1% of participants from the uncircumcised group reported use of antihypertensives and anti-diabetic medications respectively. None of the participants reported use of medications for ED. These results did not present any significant difference between the two groups (*U* = 26932.00, *Z* = 0.99, *p* = 0.318).

## Discussion

The results of this study showed higher average EF scores in circumcised men compared to uncircumcised men. The prevalence of ED was correspondingly lower in circumcised participants than in uncircumcised ones. No difference was observed in the prevalence of ED between those circumcised in childhood and those circumcised in adulthood. There were no statistically significant differences between the groups in age, relationship status, smoking, alcohol use and medication use. However, a significant difference was observed in the education level category, which demonstrated more participants with higher levels of education in the circumcision group.

The higher IIEF-5 scores that were observed in the circumcised group implied that circumcision did not have significant adverse effects that could have worsened participants’ EF. Again demographic characteristics of the two groups that were being compared were similar and only differed in the education level category, where circumcised men indicated higher education levels than their counterparts in the uncircumcised group. The observed differences in education levels between the two groups could not have had much impact on study results, as research assistants were at hand to help participants with difficulties in completing the questionnaire.

The finding in this research that circumcision does not worsen EF replicates the findings of Collins et al.,^13^ who stated that the procedure did not appear to present any clinically important effects on EF in adults who underwent the procedure. Observed higher average IIEF-5 scores in the circumcised group present a different picture from what was observed in the study by Fink et al.^9^ in which it was suggested that circumcision appeared to worsen EF. Similarities in EF in those circumcised in childhood and in adulthood agree with the findings of Aydur.^25^

There are several possible explanations for what was observed in this study. First, even if all efforts were made to assist participants with challenges in completing the questionnaire, it seems possible that the higher education levels observed in the circumcised group might have made it easier for them to understand instructions in the questionnaire and to answer them more objectively. Participants with lower education levels might have misread the questions and provided incorrect responses. It is also plausible that the opposite might have happened, with more literate participants providing misleading responses. This is especially so because the IIEF-5 tool is subjective in nature and can be reported differently by different individuals. Even if the questionnaire had been validated in other languages and cultures, this had not been done in Zambia, and this could have affected participants’ interpretation of the tool. The other explanation for these results could be recall bias, with participants self-selecting the importance of their groups or only reporting those behaviours that they considered socially acceptable.

The clinical relevance of these findings is that they demonstrate that circumcised men have normal EF, with high average IIEF-5 scores. These findings may help clinicians to better counsel those wishing to undergo circumcision.

### Limitations

In order to strengthen internal validity of this cross-sectional survey some predictor and confounding variables such as age, sexual partner relationship status, alcohol use and smoking habits were also included in the questionnaire. The measure used (IIEF-5) is a well-known international instrument with proven reliability and discriminant validity.

The study design did not allow for making conclusions about cause and effect, and it is prone to selection and measurement bias. The convenience sampling method used to recruit participants did not allow randomisation and therefore might not be representative of the male population in Lusaka. The IIEF-5 assessment tool for this survey has never been validated in Zambia. Its primary weakness is its subjective nature and reliance on self-reporting by participants. The quota sampling that was used to select some participants in the circumcised group was prone to recall bias in favour of reporting only socially acceptable outcomes. Circumcision status was not verified through physical examination. Literacy levels also differed, and this can lead to poor understanding of instructions in the IIEF-5 questionnaire, resulting in misleading responses.

One area that remains to be explored is the response of female partners of circumcised and uncircumcised men to gauge their assessment of their partners’ EF. Considering that the IIEF-5 questionnaire was applied for the first time in Zambia, there is a need to validate it locally before using it for future studies.

## Conclusion

The findings of this study show that circumcision does not confer adverse effects that could cause ED in men. These results suggest that circumcision can be considered safe in terms of EF. However, a definitive prospective study in a similar cultural context is needed to confirm these findings.
